# Increasing the HIV testing among MSM through HIV test result exchange mechanism: study protocol for a cluster randomized controlled trial

**DOI:** 10.1186/s12879-021-06484-y

**Published:** 2021-08-06

**Authors:** Yuning Shi, Jialing Qiu, Qingling Yang, Tailin Chen, Yongheng Lu, Sha Chen, Xiaoru Fan, Zhiye Lin, Zhigang Han, Jie Lu, Haobing Qian, Jing Gu, Dong Roman Xu, Yuzhou Gu, Chun Hao

**Affiliations:** 1 Department of Medical Statistics and Epidemiology, School of Public Health, Sun Yat-Sen University, Guangzhou, 510080 Guangdong P. R. China; 2 Sun Yat-Sen Global Health Institute, Institute of State Governance, Sun Yat-Sen University, Guangzhou, 510080 Guangdong P. R. China; 3 Kangyuan Community Support Center of Yuexiu District, Guangzhou, 510000 Guangdong P. R. China; 4 Guangzhou Center for Disease Control and Prevention, Guangzhou, 510440 Guangdong P. R. China; 5 Department of Health Management and Policy, College of Public Health, The University of Iowa, Iowa, IA USA; 6 Department of Health Management, School of Health Management of Southern Medical University, Guangzhou, 510000 Guangdong P. R. China; 7 ACACIA Labs, SMU Institute for Global Health and Dermatology Hospital of Southern Medical University, Guangzhou, 510000 Guangdong P. R. China

**Keywords:** HIV testing, Men who have sex with men, Online exchange mechanism, Cluster randomized controlled trial, Implementation science

## Abstract

**Background:**

HIV testing is an essential gateway to HIV prevention and treatment thus controlling the HIV epidemic. More innovative interventions are needed to increase HIV testing among men who have sex with men (MSM) since their testing rate is still low. We proposed an online HIV test results exchange mechanism whereby the one without a certified online HIV report will be asked to test HIV for exchanging HIV report with others. The exchange mechanism is developed as an extension to the existing online HIV testing service system. Through the extended system, MSM can obtain certified online HIV reports and exchange their reports with friends via WeChat. This study aims to assess effectiveness of the exchange mechanism to increase the HIV testing rate among MSM.

**Methods:**

This study will use a cluster randomized controlled trial (RCT) design. Participants are recruited based on the unit of individual social network, the sender and the receivers of the HIV report. An individual social network is composed of one sender (ego) and one or more receivers (alters). In this study, MSM in an HIV testing clinic are recruited as potential egos and forwarded online reports to their WeChat friends voluntarily. Friends are invited to participate by report links and become alters. Ego and alters serve as a cluster and are randomized to the group using the certified online HIV report with exchange mechanism (intervention group) or without exchange mechanism (control group). Alters are the intervention targeting participants. The primary outcome is HIV testing rate. Other outcomes are sexual transmitted infections, sexual behaviors, HIV testing norms, stigma, risk perception and HIV report delivery. The outcomes will be assessed at baseline and follow-up questionnaires. Analysis will be according to intention to treat approach and using mixed-effect models with networks and individuals as random effects.

**Discussion:**

This is the first study to evaluate the effectiveness of an HIV test result exchange mechanism to increase the HIV testing among MSM. This assessment of the intervention will also provide scientific evidence on other potential effects. Findings from this study will yield insights for sustainability driven by communities' intrinsic motivation.

*Trail registration:* ClinicalTrials.gov NCT03984136. Registered 12 June 2019.

**Supplementary Information:**

The online version contains supplementary material available at 10.1186/s12879-021-06484-y.

## Background

Men who have sex with men (MSM) are bearing a disproportionate burden of the global HIV epidemic, with 23% of new HIV infection cases transmitted by gay men and other MSM globally [1]. The situation is similar in China, MSM accounted for 25.5% of new HIV infections [2], and MSM infection rates in China have risen from 0.9% in 2003 to 6.9% in 2019 [3]. HIV testing is one effective way to control HIV infection, which could detect people living with HIV/AIDS and link them to antiretroviral therapy and therefore reduce HIV transmission [4]. In 2014, the Joint United Nations Programme on HIV/AIDS (UNAIDS) promoted the 90-90-90 targets to control the global HIV epidemic. Raising HIV testing uptake and frequency among concentrated populations such as MSM is critical to achieving the first 90-90-90 targets of 90% of all people living with HIV knowing their HIV status [5]. There is evidence that HIV testing in a higher frequency has been associated with lower HIV risk, and is an effective scheme to reduce the HIV infection rate among MSM [6, 7].

However, it is estimated that nearly 25% of MSM living with HIV in the United States were unaware of their infection and 33% in Europe [8, 9]. The testing rates in the above areas are close to the 90% goal but gaps persist, while it is worse in low-and-middle-income countries (LMIC), including China. In China the rate remains low despite a national HIV prevention plan targeting HIV testing for MSM has implemented [10]. Only 38% of Chinese MSM had been tested in the past year and about half of MSM had not been tested for HIV were never tested in their lifetime [11]. More efforts to promote HIV testing are needed to address the poor testing rate among MSM.

To date, there are many interventions that expand the coverage of HIV testing in MSM populations, such as Internet-based interventions that deliver health information through website or mobile applications [12–18], peer-led interventions that recruit MSM peer to transfer intervention information [19–23], community-based interventions that promote large-scale health education in community level [24–26], self-testing [27–32], etc. However, most strategies are targeting health education, mainly to raise the awareness for early HIV testing. Health education, especially the top-down perspective, has been criticized for its preaching style and lack of target population insight [33, 34]. Other strategies like HIV self-testing decrease the barriers to HIV testing uptake but are not able to motivate those who think it is unnecessary to have HIV testing. More importantly, the transmission of existing interventions mainly relies on external incentives, not the internal demand-driven. This means that these interventions may gain satisfactory outcomes during the research period, but become unsustainable after manpower input is terminated. MSM community demand-driven intervention to promote uptake of HIV testing is very few globally.

Disclosure of HIV status before sex is the MSM community spontaneous HIV risk reduction effort [35, 36]. Previous studies reported that MSM tend to selectively engage in anal intercourse with those of the same HIV status [37, 38]. However, the oral notification of one’s HIV status in daily life is not always reliable unless MSM have more convincing ways to inform their sex partners about HIV testing results. Moreover, disclosure of HIV status is not bilateral sometimes if one of them has not gotten HIV tested before. From the perspective of the social exchange theory, an individual's profitable resources are under the control of some other actors, and the pursuit of interests leads the individual to exchange resources with other actors [39]. For the MSM population, knowing sex partners’ HIV status is profitable in reducing the risk of HIV infection. In order to achieve safe sex, MSM disclose and exchange HIV test results with sex partners. When one MSM is unknown about his HIV status, the demand of exchanging the HIV test result with sex partners would drive him to HIV testing.

Based on these considerations, we proposed an online HIV test results exchange mechanism whereby the one without a certified online HIV report will be asked to take the HIV testing in exchange for the other’s certified online HIV report. Introducing HIV test results exchange mechanism into the intervention design not only meets the safe sex need of MSM, but also allows the intervention voluntarily transmitted among MSM, thus effectively and sustainably expands the coverage of HIV testing in this population. To the best of our knowledge, this is the first intervention using the HIV test results exchange mechanism in the world, which is driven from the community demand to promote HIV testing behaviors. We will evaluate the HIV test results exchange mechanism in a two-arm cluster randomized controlled trial (RCT). The primary aim of this study is to test the effectiveness of the intervention to promote HIV testing among MSM. A cluster RCT is used because the HIV test result exchange process occurs in an individual and his friends and the observations can be correlated in these MSM.

## Methods

### Study design

**Fig. 1:**
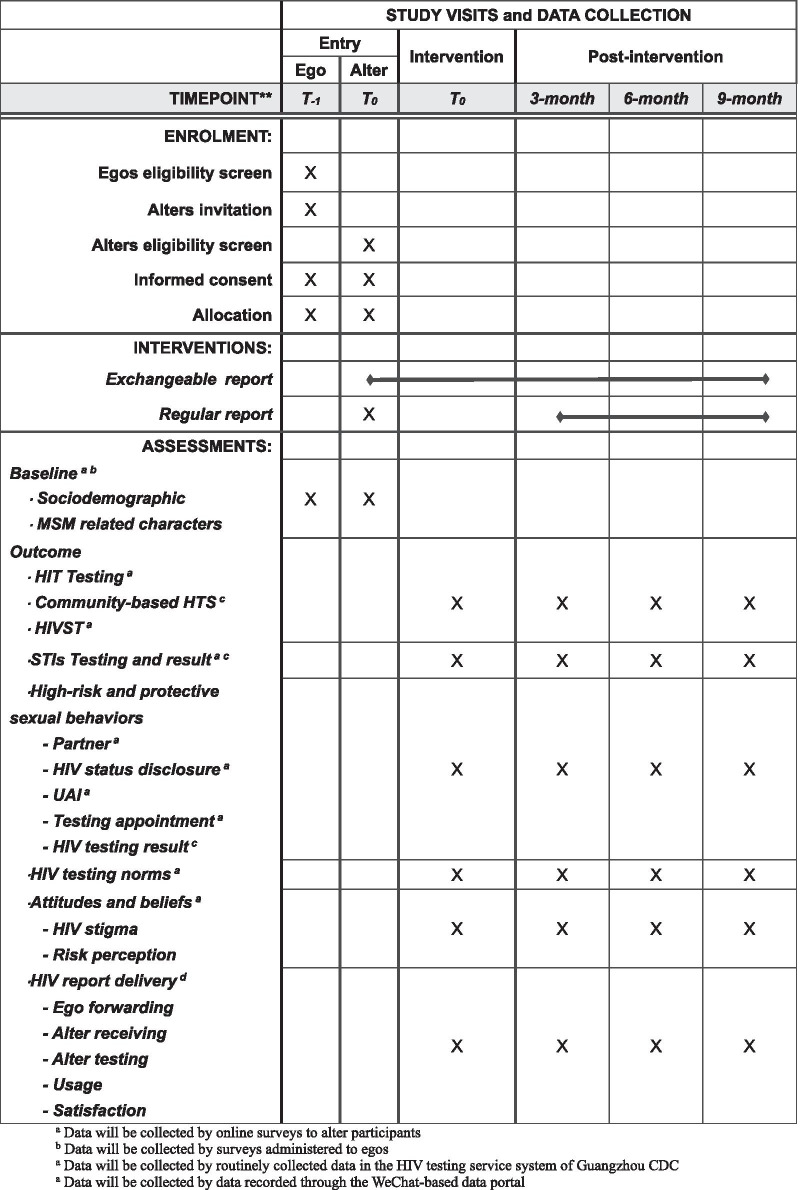
The schedule of enrolment, interventions, and assessments

A two-arm, cluster randomized controlled trial is used to evaluate the effectiveness of the HIV test results exchange mechanism on increasing HIV testing behaviors among MSM. The exchange mechanism will be developed in WeChat mini program as an extension to the existing Center for Diseases Control and Prevention (CDC) certified HIV testing service system. HIV testing, other sexual related behaviors and HIV report delivery among MSM who receive certified online HIV results (COHIV) with exchange mechanism (intervention group) and those who receive COHIV (control group) will be compared at baseline, 3, 6 and 9 months (Fig. 1).

### Study setting

#### HIV testing service setting among MSM in Guangzhou

The study will be conducted in Guangzhou, belongs to Guangdong-Hong Kong-Macao Greater Bay Area, which is located in South China. Guangzhou is one of four mega-cities in China, with 15 million population, attracts MSM in China with its tolerant environment towards homosexuality. Because of its forward economic rise, it emerged the earliest internet-based MSM dating in China. The internet-based dating website in Guangdong started from the GZTZ.ORG since 1998, and was run by this MSM community-based organization, Guangzhou Lingnan Community Support Center since 1998 [33–35].

This study will be conducted based on a famous gay-friendly HIV testing service clinic in Guangzhou, which is operated by Guangzhou Lingnan Community Support Center and supported by Centers for Disease Control and Prevention (CDC) in Guangzhou. Almost 60% of HIV testing among local MSM is conducted by this clinic every year (average 10,000 person-times per year).

#### Current WeChat-based HIV testing service system

Besides the above clinic-based testing services, a well-established WeChat mini-program of HIV Testing Service System is developed by this community organization and Guangzhou CDC, serving MSM to make appointments, register and read results report [40, 41].

WeChat was initially invented as an instant messaging app in 2011 and later became a ubiquitous daily use app integration with multi-function for local services [42]. With the growing users of WeChat in China, the number of monthly active users is over 1200 million, the WeChat-based service system was developed in 2013 [43]. Mini-programs are the “sub-applications” within the WeChat ecosystem [44], which has several benefits. Participants will get a native app-like experience without leaving WeChat interface, which enhances user convenience for swift interactions, occupies lower memory space of their smartphones, and enables convenient mini-program sharing through existing WeChat social networks, which provides a foundation for our intervention.

**Fig. 2:**
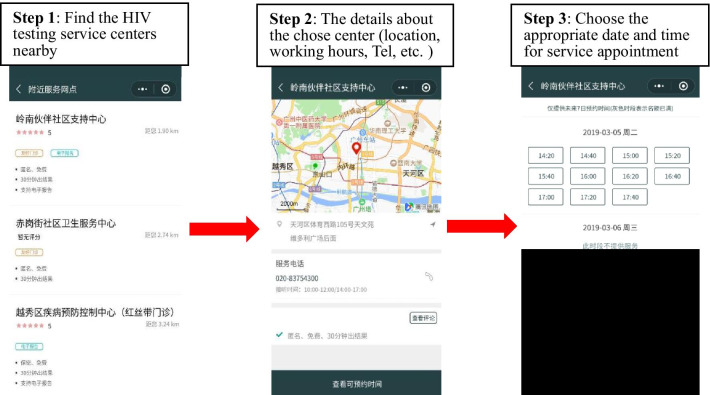
The WeChat-based HIV Testing Service System (Appointment Service module). Images depicted in this figure are screenshots of the appointment service module in the WeChat mini-program

**Fig. 3:**
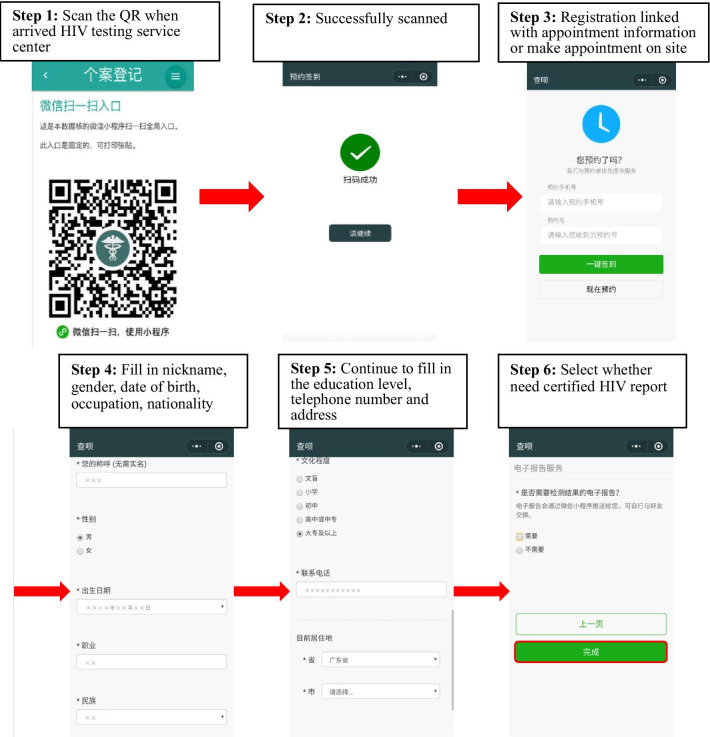
The WeChat-based HIV Testing Service System (Service Registration module). Images depicted in this figure are screenshots of the service registration module in the WeChat mini-program

**Fig. 4:**
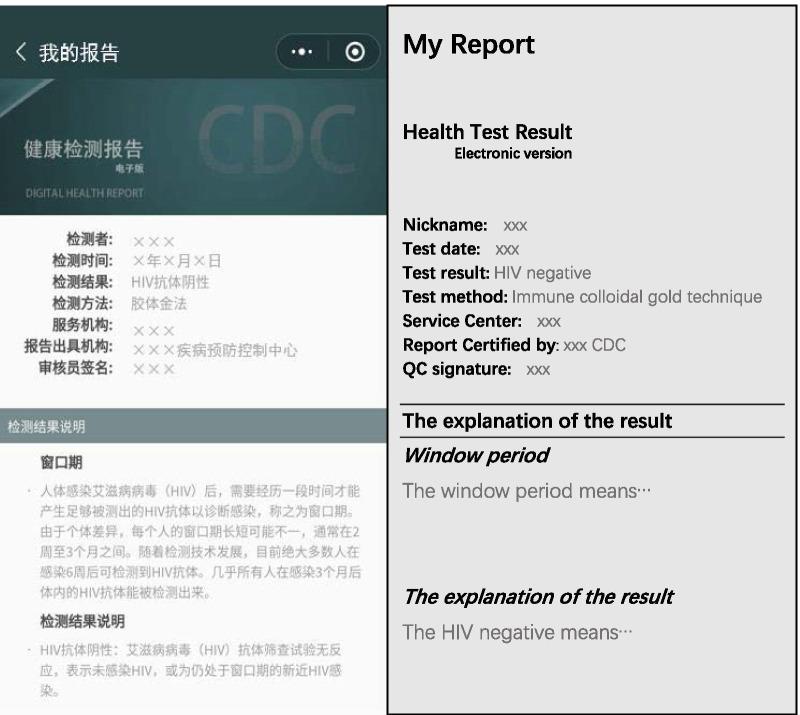
The WeChat-based HIV Testing Service System (Smartphone-based HIV Test Results Report module). The image depicted in this figure is the screenshot of the smartphone-based HIV test results report in the WeChat mini-program

Now the established WeChat-based HIV Testing Service System platform launched at the Guangzhou Lingnan Community Support Center has three modules(Wechat mini-program:查呗). The *Appointment Service* module provides nearby HIV testing service clinics’ information in users’ WeChat interfaces. MSM can choose the gay-friendly clinic and make appointments for the HIV testing service (Fig. 2). MSM arriving at the testing clinic should use WeChat to scan the QR code for registration. Then fill in the basic personal information form in the *Service Registration* module, including nickname, gender, date of birth, occupation, nation, education, phone number, address, and whether to require a certified online HIV report (Fig. 3). Only registered users can proceed to the test service. For the *Smartphone-based HIV Test Results Report Service* module, users will receive their own CDC COHIV in the mini-program after the post-test counseling. The COHIV includes the basic testing information and the test result. The former includes the tester identity (anonymous or upload a photo of the tester taken on-site), test date, test result, test method, testing site, report issuing institution, and the signature of the auditor, explanation about the meaning of the “window period” of HIV testing and the meaning of negative results (Fig. 4).

### Participants

**Fig. 5:**
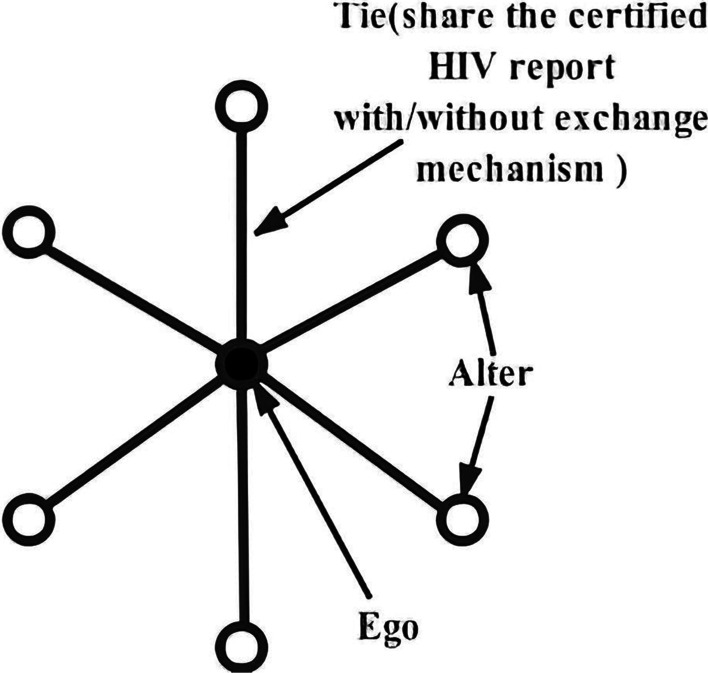
A cluster unit of egocentric social network

The potential participants are MSM who received exchangeable or regular online COHIV. The recruitment is based on the unit of an egocentric social network (Fig. 5), which includes one core member (ego) and several network members (alters) who connect to this core member. In this study, the ego is defined as one MSM people who tests HIV at the Lingnan Community Support Centre clinic and shares his COHIV via WeChat mini-program to his WeChat contactors, while alters are those contactors who receive and read the shared report. An ego and his alters are considered as a cluster in this trial. Our intervention is targeting alters, whereas egos are the recruiters of MSM alters.

#### Inclusion and exclusion criteria

Egos who are (1) aged 18 years old, (2) had anal sex with men, and (3) HIV negative (online COHIV are not available for HIV-positive individuals since there is a special legal protecting their privacy) will be included. Individuals are excluded as egos if they do not use WeChat.

The inclusion criteria of alters include (1) aged 18 years old, (2) had anal sex with men, (3) plan to live in Guangzhou in the next year, and (4) HIV unknown or negative. Individuals are excluded as alters if they cannot complete the questionnaire due to any reasons. During the post intervention stage, if a alter is tested as HIV positive, his follow-up will be terminated.

#### Egos recruitment

Egos will be recruited at the Lingnan Community Support Centre clinic. MSM who come to HIV testing and meet all the inclusion criteria will be invited as the ego during the post-test counseling. The counselor will explain the difference between HIV reports with and without exchange mechanism in detail. The MSM who agree to participate as the recruiters will complete a written consent (Additional file 1). Each ego will be randomly assigned to the intervention or control group by mini-program. The egos in the intervention group will receive the COHIV with exchange mechanism through the WeChat mini-program while the egos in the control group will receive HIV report without exchange mechanism. Egos will be informed by counselors they can share their HIV reports with friends via WeChat mini-program to recruit alters.

#### Alters recruitment

Alters recruitment will be conducted online based on the WeChat mini-program system. Alters will be reached through egos’ shared link. After an ego shares his COHIV with exchange mechanism (intervention group) or without exchange mechanism (control group) to their friends in WeChat, those who click the link will be candidate alters in our study. An online page that provides study details will be presented to the candidate alters after they read the content of ego’s report. Those interested in this study will be asked to complete the screening questionnaire and eligible individuals will fill in an online consent form (Additional file 2) and become alters.

### Randomization, blinding and allocation

Clusters will be blocked by egos and allocated 1:1 to the two groups. The random allocation sequence will be generated by using an electronic random number table and embedded in WeChat mini-program. The sequence will be concealed until the intervention is assigned. Egos will be randomly and automatically assigned to the intervention or control group by the mini-program once the online certified HIV report is sent to their WeChat. As a cluster, the group assignment of an alter is determined by his ego. This will be an open-label study in view of the intervention nature, while the statistician and the clinic staff are blinded. Contamination will be avoided by technical means in which alters in one group cannot open the shared link from egos in the opposite group.

### Intervention

#### Intervention group with exchangeable COHIV

**Fig. 6:**
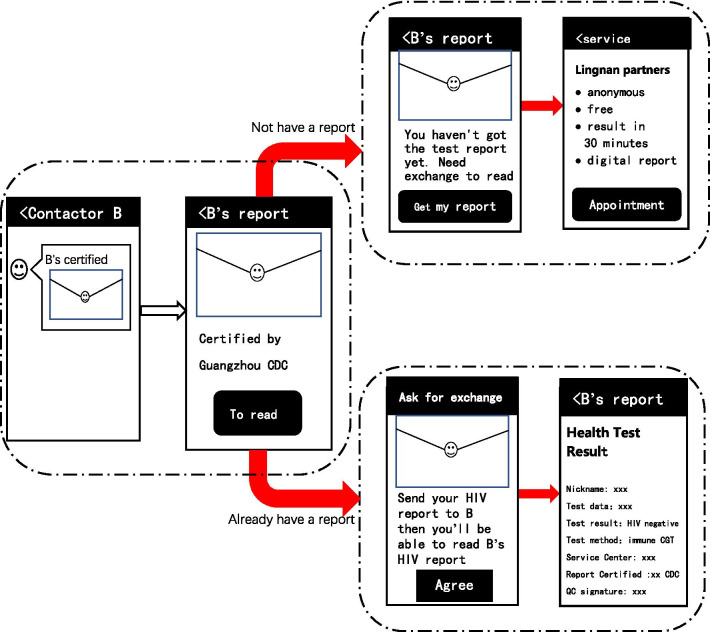
The procedure of alters in the intervention group receiving the HIV report

An exchange mechanism is developed as the extension to the above-mentioned WeChat-based HIV Testing Service System platform. In the procedure of the online COHIV exchange (Fig. 6), after alter A in the intervention group clicked “B’s HIV Test Result”, a WeChat mini-program link, from his WeChat contactor B, A will see the message that “Please send your HIV Report to B, only then you will be able to read B’s HIV Report''. Alter A will be invited to make an appointment for the HIV testing service, and linked to the *Appointment Service* module (Fig. 2). If A has the WeChat-based online COHIV, he will be able to see B’s result after sending his own and vice versa. Since there is no time frame for COHIV exchange, the most recent COHIV will be exchange between A and B if both sides have it. The most recent test date is on the COHIV. We hypothesize that MSM will be motivated to get tested if he is interested in sender as well as the sender's HIV result.

A user testing was conducted to optimize the design of the exchange mechanism. MSM were invited to provide their feedback of the mini-program, including problems encountered in the daily use of the exchange mechanism and suggestions on improving user experience of navigating between pages of the program. The wording and length of the baseline questionnaire were also checked by MSM to improve the readability of the questionnaire. Necessary modification was made during this period.

#### Control group with regular COHIV

**Fig. 7:**
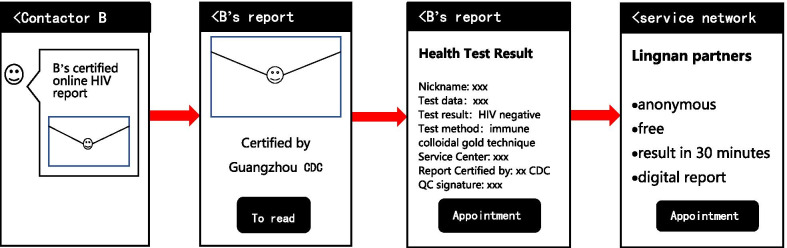
The procedure of alters in the control group receiving the HIV report

The regular COHIV without exchange mechanism is as usual service. Based on the established WeChat-based HIV Testing Service System (Figs. 2, 3, 4), MSM can share their COHIV (Fig. 4) with any WeChat contactors. Alters in the control group will see the content of the regular COHIV directly after they click the result shared by the ego. At the end of the HIV test result, the system will guide alters about how to get an HIV test by arranging appointments with a MSM friendly clinic and link to the WeChat-based *Appointment Service* module (Fig. 2). The procedure of alters in the control group receiving the certified HIV result is shown in Fig. 7.

### Outcomes and data collection

The following outcomes are collected at the time of intervention and 3, 6, 9 months in the post-intervention period. Data will be collected every three months by four ways: (1) online surveys to alter participants; (2) surveys administered to egos; (3) routinely collected data in the HIV testing service system of Guangzhou CDC; (4) data recorded through the WeChat-based data portal (Fig. 1). Online surveys to alters include baseline and follow-up questionnaires. The baseline questionnaires are assigned to alters when they click egos’ reports via the WeChat mini program. The 3, 6, 9 months post-intervention follow-up questionnaires on alters will be carried out and audited via the survey website named Wenjuanxing and via WeChat. If a questionnaire has paradoxical answers, researchers will check the answers with the alter via WeChat. Incentives (Red Envelopes) on WeChat will be given to alters every time when they finish answering and checking questionnaires. Alters will receive 50 RMB(≈ 7.5 USD) for the baseline survey, and then receive 50 RMB(≈ 7.5 USD) for the first follow-up, 20 RMB to 100 RMB(≈ 3 USD to 15.5 USD) randomly for the second and third follow-ups respectively.

The primary outcome is HIV testing in the past three months, including any self-reported HIV testing. This indicator is measured through the alters’ self-reports from the online surveys. Other outcomes include:

- HIV self-testing (HIVST) in the past three months. This indicator is measured by self-reported data from the online surveys on alters.
- Clinic-based HIV testing services (HTS) in the past three months. This indicator is calculated by service clinics that routinely collect data in the HIV testing service system of Guangzhou CDC.
- Sexual transmitted infections (STIs) related information using alters self-reports and registered data in the HIV testing system: (1) STIs testing in the past three months; and (2) STIs test result in the past three months.
- Sexual behaviors in the past three months: (1) Regular or casual sex partners; (2) request for disclosure of HIV status before sex in the past 3 months; (3) unprotected anal intercourse (UAI) with sex partners in the past 3 months. (4) HIV testing service appointment. These behaviors are recorded by the online questionnaires. (5) HIV test results. This indicator is calculated by the routinely collect data in the HIV testing service system.
- HIV testing norms [45] are measured using six survey items that are each on a four-point Likert scale in online surveys. All six items were rated on a Likert-type scale (1 = Strongly Disagree; 4 = Strongly Agree). The mean score is reported, ranged from 1 to 4. Higher values indicate less positive HIV testing social norms.
- HIV stigma [46] is measured by a 7-item version of the HIV stigma scale, designed to measure the extent to which participants anticipated negative interpersonal and interpersonal consequences were they to contract HIV in the future. All seven items were rated on a Likert-type scale (1 = Strongly Disagree; 4 = Strongly Agree). The mean score is reported, ranged from 1 to 4. Higher values indicate greater stigma.
- Risk perception of HIV in MSM community. This indicator is calculated through the online surveys.
- HIV report delivery: (1) the proportion of Egos who forward and read their reports; (2) the proportion of Alters who receive and read egos’ reports; (3) the proportion of alters who get their own HIV reports. (4) The proportion of forwarded report usage among MSM and (5) the satisfaction of online HIV report service. These data will be collected by the WeChat-based data portal.

### Sample size and statistical power

We calculated our sample size using intracluster correlation, number of outcomes, expected effect size, and the power of the study. We assumed that the intracluster correlation coefficient (ρ) was 0.2, that each social network will consist of no less than 2 alters, and that the HIV testing rate among alters in the control group during the three-month follow up will be 20%. Setting the acceptable level of significance at 0.05 and expected effect size as 10%, 176 social networks (176 egos and 352 alters) are needed in each group to identify the effect with 80% power. Assuming 30% loss of follow-up, we plan to recruit 252 egos and 504 alters in each group.

### Statistical analysis

Intent-to-treat analysis will be used to compare the outcome as well as the baseline characteristics of participants between the two groups. For the analysis of primary and secondary outcomes, mixed effect models with networks and individuals as random effects will be used to account for the clustering effect. In the main analysis, the fixed effects for the intervention group, visit number, the intervention group-by-visit number interaction will be included in the model without covariates adjustment. Appropriate link function and distribution will be used. The consistency of treatment effects on the primary outcome will be explored in predefined subgroups, including age, education, level of HIV stigma, number of COHIV received by alter, whether the alter received COHIV from his sexual partner, whether the alter exchanged COHIV with the ego, whether the alter received COHIV screenshot, whether the alter had casual partners, whether the alter had UAI with casual partners.

In the supplementary analysis, models with adjustment for age, education, UAI in the past three months, and other HIV-related health promotion received in the past three months will be performed. Secondly, models with missing data imputation will be also conducted. Missing data will be imputed using the multiple imputation approach. The above analyses are used to compare with the main analysis result to determine the robustness of the intervention effect. All the tests will be conducted using 2-sided tests at 0.05 level of significance.

### Ethical considerations

Ethics approval for this study has been obtained from Sun Yat-sen University. Informed consent for each ego will be obtained at the clinic in person, and those for each alter will be obtained online through the WeChat-based system. The study data will be collected and stored in the secure servers of Guangzhou CDC HIV testing service system, WeChat-based database and Wenjuanxing, which can be access only to the research team.

## Discussion

This trial is designed to evaluate the effectiveness of the HIV test results exchange mechanism in contributing to HIV testing among a high HIV-hazardous but exclusive population, MSM. Existing interventions designed to increase HIV testing among MSM are mostly restricted to health education and relied on external promotion. In this study, the intervention is HIV test results exchange mechanism and MSM are motivated to require HIV testing to exchange online COHIV with his friends. Innovative aspects of this intervention are that the exchange mechanism (1) internally generates from MSM’s need to know sex partner’s HIV status before sex, (2) is designed as an online intervention embedded in the most popular chat app in China, and (3) will be spontaneously transmitted by MSM through their social networks. Moreover, the exchange mechanism is similar to disclosure of HIV status in daily life but the COHIV exchange process is designed as compulsive and extended the intervention to the Internet platform. These characteristics mentioned above stand that the exchange mechanism can overcome the geographic distance and reach a larger population as an mHealth intervention. Further, this intervention will face less obstacles about long-term sustainability since it is spontaneously transmitted by MSM and driven by their need. Although there exists a study providing online certified HIV report to MSM, the author did not explore how the certified online HIV report facilitates testing behaviors of the users [47]. To our knowledge, this study represents the first attempt to assess the effectiveness of the exchange mechanism among MSM and holds great promise for increasing the HIV testing rate of MSM in the future. With the successful implementation and more HIV testing boosted by the exchange mechanism, it will light a pathway toward broader usage of the online COHIV and exchange mechanism. Additionally, this intervention has impact on reducing the embarrassment of initiating HIV-status discussion with sex partners among MSM [47].

## Limitations

Although this trial provides a new vision for maintaining sustainability of promotion of HIV testing, some limitations exist. The exchange mechanism is based on smartphones and WeChat mini-program which may create barriers to elderly MSM, while recent research shows that the age of MSM participating in a study of mobile applications is mainly 18–45 years old [48]. Besides, the study site is a community service center in Guangzhou, which may limit representativeness of other testing sites such as the government service center and sexual diseases clinics and lead to sampling bias and restrict the generalizability of results.

Further qualitative interviews can be conducted to maximize the identification of health impacts that the intervention might bring. It is more important for the qualitative research to generate in-depth explanations of specific study phenomena than provide results at a statistical level. Given the current lack of data on the exchange mechanism, we believe that qualitative interviews to identify key aspects of implementation will be essential to complement the existing knowledge base.

## Supplementary Information

**Additional file 1.** HIV testing Promotion Project Informed Consent for egos.

**Additional file 2. **HIV testing Promotion Project Informed Consent for alters.

## Data Availability

The datasets used and/or analyzed for the further study are available from the corresponding authors on reasonable request.

## Abbreviations

MSM: Men who have Sex with Men
UNAIDS: Joint United Nations Programme on HIV/AIDS
LMIC: Low-to-Middle-Income Country
RCT: Randomized Controlled Trial
CDC: Centers for Disease Control and Prevention
COHIV: Certified Online HIV results
UAI: Unprotected Anal Intercourse
HTS: Community-based HIV Testing Services
HIVST: HIV Self-Testing
STIs: Sexual Transmitted Infections
