# Supplementary material for: Increasing the HIV testing among MSM through HIV test result exchange mechanism: study protocol for a cluster randomized controlled trial
Source: BMC Infect Dis. 2021 Aug 6;21:764. doi: 10.1186/s12879-021-06484-y (PMC8343929; doi:10.1186/s12879-021-06484-y)
Supplement: Supplementary file 1 — Additional file 1. HIV testing Promotion Project Informed Consent for egos. [file 12879_2021_6484_MOESM1_ESM.docx]

**HIV Testing Promotion Project Informed Consent**

Hello!

The School of Public Health of Sun Yat-sen University is currently conducting an evaluation of HIV testing promotion in the gay community. The purpose of this project is to assess whether online HIV test results reports can promote the behavior of gay men in HIV testing. The findings will provide evidences to community organizations and health authorities to improve gay community health services and the overall health of gays. We sincerely invite you to participate in this program, which is closely linked to your own health. In this project, the sections you need to assist or understand including:

1. You can obtain a CDC-certified online HIV testing reporting service after testing, which can be shared with your friends based on certain rules.

2. During the project, from September 2019 to September 2021, you may not receive an online HIV testing report forwarded by some of your friends due to research needs.

Whether or not you would like to participate in this program is completely voluntary and will not have any adverse effects on you, and if you do not wish to participate, you may not accept it. If you fully understand the purpose and content of this survey and agree to participate in this program, please sign this consent form. You can keep a copy of this consent. If you have any questions about this survey, please contact the project leader, Professor Chun Hao (tel:020-87334892).

Nickname: Agree ( ) Disagree ( )

Investigator's Signature: Investigation Date:

1. What type of online report would you prefer to use? (Single-choice questions)

| 🞎 [Exchange to read type], that is, I send it to others, the receiver need to send me his report back, in order to see my test results. |  |
| --- | --- |
| 🞎 [Direct to read type], that is, I send it to others, the receiver can see my test results directly. |  |
| 🞎 Either of the above is fine |  |

2. In Scenarios 1 to 4, which of the following friends might you send your report to? (Multi-choice questions)

|  | | Scenario 1:  My report is [Exchange to read type] | Scenario 2:  My report is [Direct to read type] | Scenario 3:  My report is [Exchange to read type] and [Red envelop Incentive Forwarding] | Scenario 4:  My report is [Direct to read type] and [Red envelop Incentive Forwarding] |
| --- | --- | --- | --- | --- | --- |
| I'll send the report to | Regular sex partner (boyfriend or regular sex partner). | 🞎 | 🞎 | 🞎 | 🞎 |
|  | Friends in the circle | 🞎 | 🞎 | 🞎 | 🞎 |
|  | Casual sex partner (one-night stand) | 🞎 | 🞎 | 🞎 | 🞎 |
|  | A stranger who I intend to develop a romantic relationship or a regular partner with | 🞎 | 🞎 | 🞎 | 🞎 |
|  | Others | 🞎 | 🞎 | 🞎 | 🞎 |
| I probably won't send my own report to anyone else | | 🞎 | 🞎 | 🞎 | 🞎 |

The person who will forward reports please fill in the following question

3.1. Do you think the exchange of online reports can help you understand the infection status of your sexual partner? ( Single-choice questions)

| 🞎Almost none |
| --- |
| 🞎Less |
| 🞎Ordinary |
| 🞎More |
| 🞎Very much more______________ |

People who can't forward fill in the following two questions

3.2.1 In question 16/17/18/19, why don't you forward your report? [Fill in the blanks]

_________________________________

3.2.2 What do you think can help you forward your report? (e.g. cash prizes / prizes, etc.) [Fill in the blanks]

_________________________________
