# Supplementary material for: Increasing the HIV testing among MSM through HIV test result exchange mechanism: study protocol for a cluster randomized controlled trial
Source: BMC Infect Dis. 2021 Aug 6;21:764. doi: 10.1186/s12879-021-06484-y (PMC8343929; doi:10.1186/s12879-021-06484-y)
Supplement: Supplementary file 2 — Additional file 2. HIV testing Promotion Project Informed Consent for alters. [file 12879_2021_6484_MOESM2_ESM.docx]

**HIV testing Promotion Project Informed** **Consent**

Hello!

The School of Public Health of Sun Yat-Sen University is currently conducting an evaluation of HIV testing promotion in the gay community. The purpose of this project is to assess whether online HIV test results reports can promote the behavior of gay men in HIV testing. The findings will provide evidences to community organizations and health authorities to improve gay community health services and the overall health of gays. We sincerely invite you to participate in this program, which is closely linked to your own health. In this project, the sections you need to assist or understand including:

1. You can make an appointment of HIV testing through our WeChat mini-program, after testing you can obtain HIV testing online report. The report has a forwarding function, that is, you can forward your detection electronic report to your WeChat friends, but the report is forwarded to follow certain rules.
2. During the project, from August 2019 to August 2020, you may not receive some of the HIV test online reports forwarded by your WeChat friends due to research needs.
3. Assist in completing the 3-monthly online questionnaire (4 times in total), costing 5-10 minutes every time: From now on, you will receive an invitation to an online survey every 3 months, including this one, 3 months later, 6 months later and 9 months later, four times in total. Each time takes 5-10 minutes. The purpose of the online surveys is to understand your HIV testing from the time you receive the online test report to assess the effectiveness of the intervention project. In addition to HIV testing, the questionnaire will also ask you for some background information and other aspects of your health, which may involve some of your privacy. The primary purpose of these information we collect is to more accurately assess the effectiveness of interventions. The survey will be conducted in anonymous. In addition, all investigations are confidential throughout the investigation and will never be disclosed to anyone else without your consent.

Whether or not you would like to participate in this program is completely voluntary and will not have any adverse effects on you, and if you do not wish to participate, you may not accept it. If you fully understand the purpose and content of this survey and agree to participate in this program, please sign this consent form. Once each questionnaire is completed and confirmed by the staff member, you will receive the appropriate reward as compensation for the time you have spent. When you complete the first questionnaire, you will receive a 50 RMB(≈7.5 USD) WeChat red envelope, when you complete the second questionnaire, you will receive a 50 RMB(≈7.5 USD) WeChat red envelope, after completing the third questionnaire, you will receive a 20 RMB to 100 RMB(≈3 USD to 15.5 USD) WeChat red envelope randomly, and after completing the fourth questionnaire, you will also receive a 20 RMB to 100 RMB(≈3 USD to 15.5 USD) WeChat red envelope randomly. You can keep a copy of this consent. If you have any questions about this survey, please contact the project leader, Professor Chun Hao (tel:020-87334892).

Respondent number: Agree ( ) Disagree ( )

Investigator's Signature: Investigation Date:
